# Empagliflozin alleviates renal inflammation and oxidative stress in streptozotocin-induced diabetic rats partly by repressing HMGB1-TLR4 receptor axis

**DOI:** 10.22038/ijbms.2019.31788.7651

**Published:** 2019-04

**Authors:** Zahra Ashrafi Jigheh, Amir Ghorbani Haghjo, Hassan Argani, Leila Roshangar, Nadereh Rashtchizadeh, Davoud Sanajou, Saeed Nazari Soltan Ahmad, Jalil Rashedi, Siavoush Dastmalchi, Mehran Mesgari Abbasi

**Affiliations:** 1 Department of Biochemistry, Faculty of Medicine, Tabriz University of Medical Sciences, Tabriz, Iran; 2 Biotechnology Research Center, Tabriz University of Medical Sciences, Tabriz, Iran; 3 Urology and Nephrology Research Center, Beheshti University of Medical Sciences, Tehran, Iran; 4 Stem Cell Research Center, Tabriz University of Medical Sciences, Tabriz, Iran; 5 Drug Applied Research Center, Tabriz University of Medical Sciences, Tabriz, Iran

**Keywords:** Diabetic nephropathy, Empagliflozin, HMGB1, Inflammation, TLR-4

## Abstract

**Objective(s):**

Empagliflozin, a sodium-glucose cotransporter-2 (SGLT-2) inhibitor, possesses verified anti-inflammatory and anti-oxidative stress effects against diabetic nephropathy. The present investigation aims to examine empagliflozin effects on the renal levels of high mobility group box-1 (HMGB1), a potent inflammatory cytokine, and its respective receptor toll-like receptor-4 (TLR-4) in STZ-induced diabetic rats.

**Materials and Methods:**

Empagliflozin at 10 mg/kg per os (p.o.) was administered for 4 weeks, starting 8 weeks after the induction of diabetes. Renal function, kidney inflammation, oxidative stress, and apoptosis markers as well as renal HMGB1, receptor for advanced glycation end products (RAGE), and TLR-4 levels were assessed.

**Results:**

In addition to down-regulating NF-κB activity in renal cortices, empagliflozin reduced renal levels of HMGB1, RAGE, and TLR-4. It alleviated renal inflammation as indicated by diminished renal expressions of inflammatory cytokines and chemokines like tumor necrosis factor-alpha (TNF-α) and monocyte chemoattractant protein-1 (MCP-1) and also decreased urinary levels of interleukin-6 (IL-6) and alpha-1 acid glycoprotein (AGP). Moreover, empagliflozin ameliorated renal oxidative stress as demonstrated by decreased renal malondialdehyde (MDA) and elevated renal activities of superoxide dismutase (SOD) and glutathione peroxidase (GPX). It also suppressed renal caspase-3, the marker of apoptosis; and furthermore, enhanced renal function noticed by the declined levels of serum urea and creatinine.

**Conclusion:**

These findings underline that empagliflozin is able to attenuate diabetes-related elevations in renal HMGB1 levels, an influential inflammatory cytokine released from the necrotic and activated cells, and its correspondent receptors, i.e., RAGE and TLR-4.

## Introduction

Diabetic nephropathy, one of the most prevalent complications of diabetes mellitus, is the main cause of end-stage renal disease (ESRD) worldwide (1). Pathogenetically, renal proximal tubular cells are involved in the initiation and progression of diabetic nephropathy. Glucose entry into these cells occurs independently of insulin and therefore chronic glucose accumulation activates intracellular signaling pathways involved in the enhancement of inflammation, oxidative stress, and apoptosis that seriously impair renal function (2).

While the pathogenesis of diabetic nephropathy has traditionally been attributed to the binary of hemodynamic alterations and severe constant hyperglycemia, accumulating evidence underlines the principal contribution of inflammation and oxidative stress to the progression of the disease (3, 4). The surge in various inflammatory cytokines and chemokines including tumor necrosis factor alpha (TNF-α) and monocyte chemoattractant protein 1 (MCP-1) accompanied by the increased infiltration of the innate immune cells into the renal tissues has been demonstrated to evolve in the disease course (5). Indeed, *in vitro* investigations on the renal tubular cells have confirmed that ample amounts of glucose uptake via the sodium-glucose cotransporters-2 (SGLT-2) elicit serious oxidative stress, resulting in increased rates of apoptosis (6).

Toll-like receptor-4 (TLR-4) has been implicated in the pathophysiology of diabetic nephropathy since its elevated expressions and increased stimulation in the diabetic milieu of the kidneys highly aggravate the tubulointerstitial inflammation (7). Additionally, high mobility group box-1 (HMGB-1), a nuclear protein serving as a gene expression co-factor, when released from the activated renal cells in diabetic conditions, acts as an efficient proinflammatory molecule by stimulating the receptor for advanced glycation end-products (RAGE) and TLR-4 (8).

**Figure 1:**
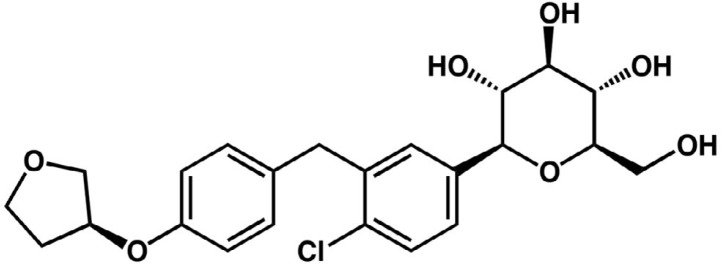
Empagliflozin chemical structure

Empagliflozin (BI 10773; 1-chloro-4-(β-D-glucopyranose-1-yl)-2-[4-((S)-tetrahydrofuran-3-yl-oxy)-benzyl]-benzene; Figure 1) is a water-soluble SGLT-2 inhibitor developed for the management of type 2 diabetes mellitus as a glucose-lowering agent (9). Furthermore, since it lowers glucose uptake by the renal proximal tubular cells, experimental *in vivo* studies have shown its ability in suppressing renal inflammation as well as oxidative stress and in attenuating renal tubular cell injuries via inhibition of AGE/RAGE/NF-κB axis (10, 11). Considering the fundamental role of HMGB-1 and TLR-4 in the pathogenesis of diabetic nephropathy, in the present investigation, we aimed to examine the effects of empagliflozin on the renal levels of these proteins and further studied their downstream effectors in the urine and renal tissues of STZ-induced diabetic rats.

## Materials and Methods

***Chemicals***

Empagliflozin was purchased from Cayman Chemical (Ann Arbor, MI) and streptozotocin was obtained from Santa Cruz (Dallas, TX).

***Animal experiments***

Eight-week-old male Wistar rats (180-200 g) were obtained from the Animal Care Center, Tabriz University of Medical Sciences. Animals had *ad libitum* access to food and water and all experimental procedures were conducted in accordance with the instructions issued by the Council of Research and Technology, Tabriz University of Medical Sciences. Diabetes was induced by an intraperitoneal (IP) injection of STZ (50 mg/kg) in a 10 mM citrate buffer (pH 4.5) and confirmed by a tail-blood glucometer 48 hr after STZ injection; rats with blood glucose levels of 270 mg/dl or greater were considered diabetic. Animals were allocated into three groups of: 1) healthy control rats (Control, n = 8), 2) diabetic control rats (Diab, n = 8), and 3) Empagliflozin treatment rats (10 mg/kg, intra-gastric gavage) (10). Animals were housed in standard conditions for two months in order to allow renal sequelae of diabetes mellitus develop (12), and then treatment with empagliflozin (Cayman Chemical) started for a period of 4 weeks. 12-hr urine samples were collected at the penultimate day of the study by housing the animals in the metabolic cages.

Finally, a combined lethal dose of 100 mg/kg ketamine and 1 mg/kg midazolam were injected; blood collection via cardiac puncture was done; and after excising, the right kidneys were fixed in 10% formalin for histological assessments; and the left kidneys were promptly stored at -80 ^°^C for biochemical analyses.

**Table 1:** List of primers

Names of genes	Forward primer (5′ to 3′)	Reverse primer (5′ to 3′)
Fibronectin	TCAAAACCAGTTGGGGAGTC	TCAAAACCAGTTGGGGAGTC
Collagen type IV	CCATGCACCACACTTAAAGG	GCCTCTGTTTCCCTTTTCAC
TNF-α	ACCACGCTCTTCTGTCTACTG	CTTGGTGGTTTGCTACGAC
TGF-β	GCTGTCTTTTGACGTCACTG	GTTTGGGACTGATCCCATTG
CXCL12	GCTCTGCATCAGTGACGGTAAG	TGGCGACATGGCTCTCAAA
RANTES	GTCTTTGTCACTCGAAGGAAC	CAGGATCAGAATGGAGAGACC
MCP-1	GGCAAGATGATCCCAATGAG	TCTGATCTCACTTGGTTCTGG
RAGE	CAGAAACCGGTGATGAAGGAC	TCTGGGTTGTCGTTTTCGC
GAPDH	GTCGGTGTGAACGGATTTG	TCCCATTCTCAGCCTTGAC

**Table 2:** General characteristics of rats in all study groups

	Control (n = 8)	Diab (n = 8)	EMP (n = 8)
Body weight, g	343.25± 11.05	298 ± 8.33^a^	291 ± 10.88
Serum glucose, mg/dL	82.87 ± 3.31	435.57 ± 66.56^a^	205± 47.53^a^,^b^
HbA1c, %	5.5 ± 0.45	9.6 ± 0.86^a^	6.4 ± 0.71^a^,^b^
Serum urea, mg/dL	32.81 ± 7.1	57.39 ± 10.48^a^	42.11 ± 9.57^a^,^b^
Serum creatinine, mg/dL	0.67 ± 0.02	1.01 ± 0.05^a^	0.83 ± 0.01^a^,^b^

Control, healthy control rats; Diab, diabetic control rats; EMP, diabetic rats treated with empagliflozin. Values are presented as mean ± S.D. HbA1c, hemoglobin A1c.

a : *P<*0.01 vs Control rats

b : *P<*0.01 vs Diab rats

**Figure 2:**
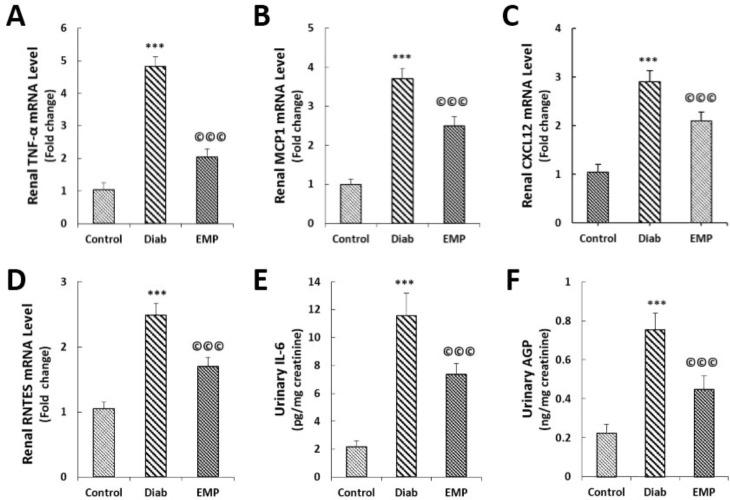
Effects of empagliflozin on renal gene expressions of TNF-α (A), MCP-1 (B), CXCL12 (C), RANTES (D), urine IL-6 levels (E), and urine AGP levels (F). A to D: Gene expressions were assessed by RT-qPCR. E and F: urinary levels of IL-6 were measured by the ECLIA method and AGP levels in urine were assayed by PETIA; data were normalized by urine creatinine levels as measured by Jaffe’s method. TNF-α, tumor necrosis factor alpha; MCP-1, monocyte chemoattractant protein 1; CXCL12, C-X-C Motif Chemokine Ligand 12; RANTES, regulated on Activation, Normal T Cell Expressed and Secreted; RT-qPCR, real-time quantitative polymerase chain reaction; ECLIA, electro-chemiluminescent immunoassay; AGP, alpha-1 acid glycoprotein; PETIA, particle enhanced turbidimetric immunoassay. Control, healthy control rats; Diab, diabetic control rats; EMP, diabetic rats treated with empagliflozin. A to E: ***P<0.01 vs Control group. A to E: ©©©*P*<0.01 vs Diab rats

**Figure 3:**
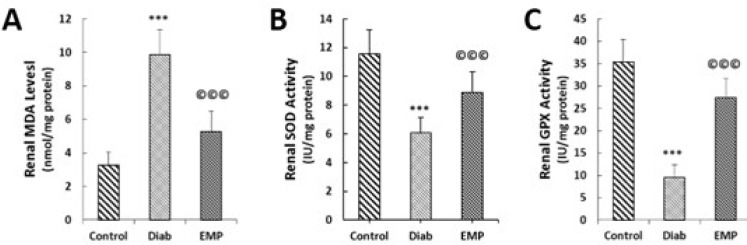
Effects of empagliflozin on renal concentrations of MDA (A), renal SOD activities (B), and renal GPX activities (C). MDA, malondialdehyde; SOD, superoxide dismutase; GPX, glutathione peroxidase. Control, healthy control rats; Diab, diabetic control rats; EMP, diabetic rats treated with empagliflozin. ***P<0.01 vs Control group. A to C: ©©©*P*<0.01 vs Diab rats

**Figure 4:**
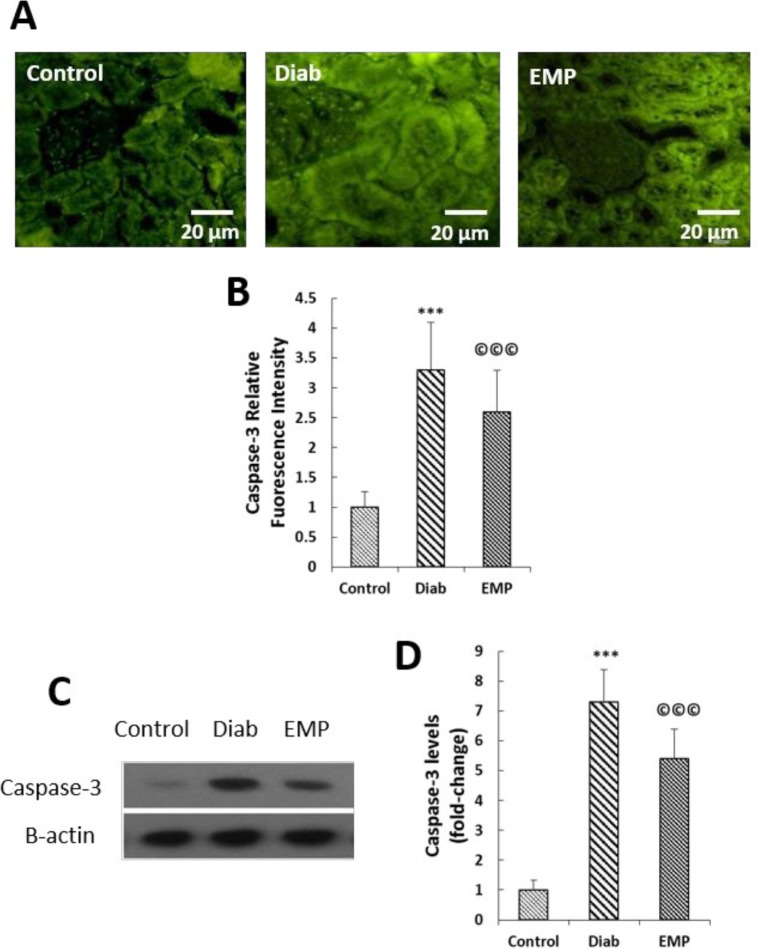
Effects of empagliflozin on renal caspase-3 levels (A to B). A and B: as assessed by Immunofluorescence analysis; and obtained data were normalized by the values of the control group. C and D: as assayed by Western blotting; the values were first normalized by β-actin intensities and then were reported related to the values of the control group. Control, healthy control rats; Diab, diabetic control rats; EMP, diabetic rats treated with empagliflozin. ***P<0.01 vs Control group. ©©©*P*<0.01 vs Diab rats

**Figure 5:**
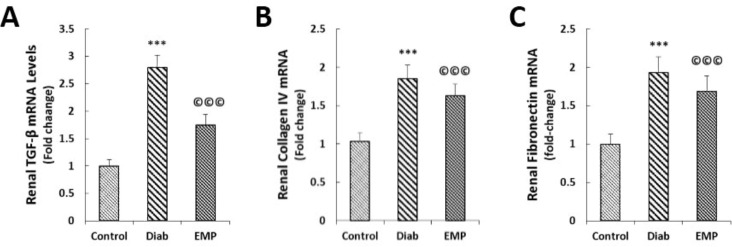
Effects of empagliflozin on renal expressions of TGF-β (A), collagen type IV (B), and fibronectin (C). A to C: Total RNAs extracted from the renal tissues of Control, Diab, and EMP rats were transcribed into complementary cDNA. Quantitative real-time PCR was conducted. Data were normalized by the intensities obtained for GAPDH house-keeping gene. TGF-β, transforming growth factor beta; GAPDH, glyceraldehyde-3-phosphate dehydrogenase. Control, healthy control rats; Diab, diabetic control rats; EMP, diabetic rats treated with empagliflozin. A to C ***P<0.01 vs Control group. ©©©*P*<0.01 vs Diab rats, respectively. n=8 for all groups

**Figure 6:**
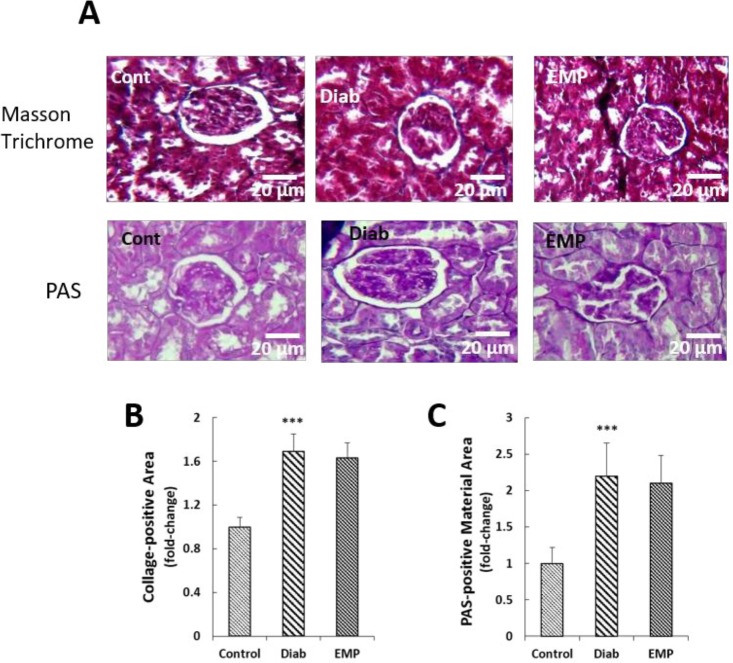
Effects of empagliflozin on collagen deposition (A) and PAS-positive material deposition (B) in renal tissues. A: Sections were stained by Masson's trichrome. B: Sections were stained by period acid Schiff. Pictures related to 20 randomly selected fields were analyzed using ImageJ software and the pixel intensities of the respective area were measured. The values in Diab and EMP groups were normalized by the values obtained for control rats. PAS, periodic acid-Schiff; Control, healthy control rats; Diab, diabetic control rats; EMP, diabetic rats treated with empagliflozin. ***P<0.01 vs Control group vs Diab rats

**Figure 7:**
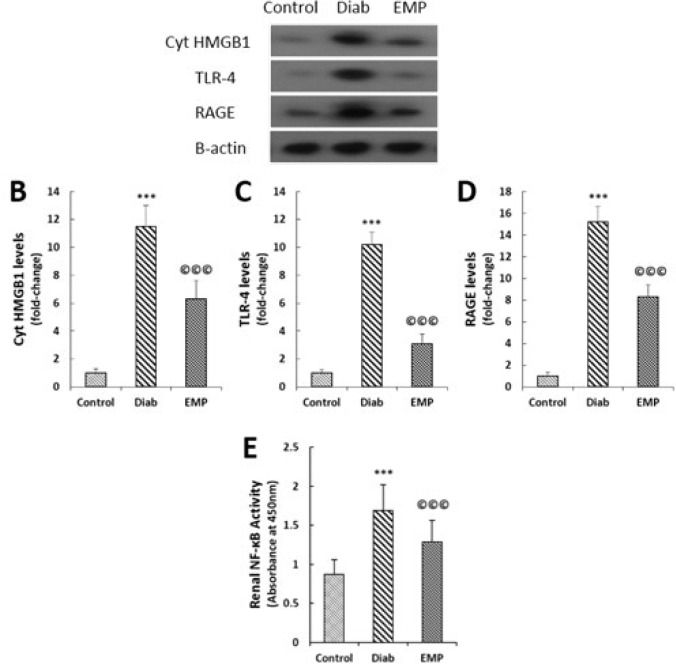
Effects of empagliflozin on renal cytoplasmic HMGB1 levels (A and B), RAGE levels (A and C), TLR-4 levels (A and D), and NF-κB activities (D). A to D: Representative bands of Western blot with semi-quantitative data. Data were normalized by the intensity of β-actin bands and then related to the values acquired by the controls. D: Nuclear fractions of tissue lysates were isolated by a nuclear extraction kit; phosphorylated NF-κB p65 subunit was detected using an ELISA kit; absorbance of the microplate wells at 450 nm was considered as the extent of NF-κB activity. HMGB1, high mobility group box 1; Cyt HMGB-1; Cytosolic HMGB-1; RAGE, receptor for advanced glycation end products; TLR-4, toll-like receptor 4; ELISA, enzyme-linked immunosorbent assay. Control, healthy control rats; Diab, diabetic control rats; EMP, diabetic rats treated with empagliflozin. ***P<0.01 vs Control rats, ©©©P<0.01 vs Diab rats

***RT-qPCR***

Total RNA was extracted from 30 mg kidney tissue using NucleoSpin RNA extraction kit (Macherey-Nagel, Ulm, Germany) based on the manufacturer’s instructions. cDNA was synthesized using the REVERTA-L RT reagents kit (InterLabServices, Moscow, Russia) according to the protocols issued by the manufacturer. RT-qPCR was performed using SYBR Green (Amplicon, Brighton, UK) detection method with Mic thermocycler (BioMolecular Systems, Upper Coomera, Australia). The primer sequences have been presented in Table 1.

***Immunofluorescence and histopathologic examinations***

5-micron thick kidney tissue sections were prepared and the antigens were enzymatically retrieved by 0.05% trypsin (Sigma-Aldrich, St. Louis, MO) after deparaffinization. Then, for indirect immunofluorescence staining, sections were incubated in the blocking reagent (SantaCruz) for 1 hr followed by washing and incubation with the caspase-3 primary antibody (SantaCruz, sc-7272) overnight at 4 ^°^C. Finally, the sections were incubated for 60 min at room temperature with the secondary FITC-conjugated antibody (SantaCruz, sc-516140) in a dark chamber; and visualized using a fluorescent microscope with appropriate filters. 20 random fields (×400) per rat were captured and the fluorescence intensities were quantified using the ImageJ picture analysis software (ver. 1.41). Fluorescence intensities of control diabetic and empagliflozin treated rats were normalized by the values obtained for healthy control ones.

To evaluate renal fibrosis, 5-μm thick tissue sections were cut and mounted on glass slides for Masson’s trichrome staining. 20 fields (×400) were randomly selected and the blue-stained collagen positive areas were quantitatively measured with ImageJ software (version 1.41). Periodic acid-Schiff (PAS) staining was also performed for the assessment of PAS-positive material deposition in the kidney sections. Pixel intensities correspondent to PAS-stained areas were measured using ImageJ (version 1.41) and the values obtained for the diabetic control and empagliflozin treatment groups were normalized by the values calculated for the healthy control group.

***Western blotting***

50 mg of kidney tissues were lysed in RIPA (radioimmunoprecipitation assay) buffer (SantaCruz) and their total protein concentrations were measured by the Lowry method to measure RAGE and TLR-4 protein levels. For the measurement of cytoplasmic HMGB1, first cytoplasmic and nuclear fractions were isolated using a nuclear extraction kit (Cayman Chemical). Separation was performed by SDS-PAGE followed by electro-blotting to transfer separated proteins onto PVDF membranes. After blocking with nonfat milk blocking solution for 60 min, the membranes were incubated in RAGE (SantaCruz, sc-365154), HMGB-1 (SantaCruz, sc-56698), TLR-4 (SantaCruz, sc-293072), and β-actin (SantaCruz, sc-47778) primary antibodies at 4 ^°^C overnight. Thereafter, blots were incubated in HRP-labeled secondary antibodies at room temperature for 45 min and were visualized by Pierce ECL Western Blotting Substrate (Thermo Fisher Scientific, Waltham, Massachusetts, USA). Band densities on the pictures obtained from x-ray films were quantitated with ImageJ software (version 1.41) and normalized by the values obtained for β-actin bands as the loading control.

***Biochemical analyses***

Blood hemoglobin A1c (HbA1c) was measured with the ion-exchange micro-column chromatography method using a commercial kit (BioSystems, Barcelona, Spain). Serum/urine creatinine levels and serum urea levels were quantified by Jaffe’s and enzymatic methods, respectively, using commercial kits (Pars Azmoon, Tehran, Iran). Serum glucose levels were assayed by the glucose oxidase method by using a commercial kit (Pars Azmoon, Tehran, Iran). Renal malondialdehyde (MDA) concentrations were measured via a colorimetric MDA assay kit (ZellBio, Ulm, Germany). Moreover, kidney superoxide dismutase (SOD) and glutathione peroxidase (GPX) activities were assayed calorimetrically using commercial kits (BiorexFars, Shiraz, Iran); The values obtained for MDA concentrations and SOD/GPX activities were normalized by the renal total protein concentrations measured with Lowry assay. A particle-enhanced turbidimetric immunoassay (PETIA) was adopted to measure urinary alpha-1 acid glycoprotein (AGP) levels (Aptec Diagnostics, Sint-Niklaas, Belgium); and electrochemiluminescent immunoassay (ECLIA) was the selected method to assay interleukin-6 (IL-6) levels in the urine (Roche Diagnostics, Basel, Switzerland). The values obtained for urinary AGP and IL-6 were normalized by urine creatinine (Cr) levels.

***NF-*** ***κB*** ***activity assay***

NF-κB p65 subunit phosphorylation levels in the nuclear fractions of renal tissues were assayed using an ELISA-based commercial kit (NF-κB (p65) Transcription Factor Assay Kit, Cayman Chemical, Ann Arbor, MI). Nuclear extracts were separated using a commercial nuclear extraction kit (Cayman Chemical) according to the manufacturer’s instructions; then, the protein contents of these nuclear extracts were quantified by Lowry assay. Appropriate amounts of nuclear extracts containing 20 μg protein were loaded into the microplate wells. The wells were coated with corresponding double-stranded DNAs only capable of binding to the phosphorylated p65 subunits. After washing steps, anti-phosphorylated p65 primary antibodies followed by HRP-conjugated secondary antibodies were applied; then, the absorbance of the wells was read at 450 nm.

***Statistical analysis***

Data are presented as means ± SD. One-way analysis of variance followed by Tukey’s *post hoc* analysis was performed for statistical comparisons; *P<*0.05 was considered significant. The analyses were performed using the SPSS software version 18 (IBM, Chicago, IL).

## Results

***General characteristics***

General characteristics of the study groups are presented in Table 2. Diabetic rats had significantly reduced weights as compared to the normal ones and treatment with EMP had no significant impact on the body weight. Remarkably increased serum glucose and blood HbA1c levels, however, were efficiently decreased by EMP. Renal function tests, i.e., serum urea and creatinine levels turned out to be increased in the control diabetic rats and EMP treatment significantly attenuated their values (Table 2).

***Renal levels of inflammatory, oxidative stress, and apoptosis markers***

As shown in Figure 1, our gene expression analysis by RT-qPCR demonstrated that EMP reduced diabetes-induced elevations in the renal expressions of pro-inflammatory cytokine and chemokines TNF-α, MCP-1, CXCL12, and RANTES (Figures 2A to 2D). Urinary markers of kidney inflammation, i.e., AGP and IL-6 were noticed to be significantly elevated in the rats of the control diabetic group, and EMP efficiently decreased their levels in the urine (Figures 2E and 2F). Additionally, renal concentrations of MDA were mitigated and at the same time, renal activities of SOD and GPX were elevated after treatment with EMP (Figures 3A to 3C). We also measured renal levels of the apoptosis marker caspase-3 by immunofluorescence and Western blotting methods. Our findings revealed that the increased levels of caspase-3 protein in control diabetic rats were significantly reduced after treatment with EMP for 4 weeks (Figures 4A to 4D).

***Assessment of renal fibrosis***

EMP lowered diabetes-related increases in the renal expressions of pro-fibrotic gene TGF-β and the fibrotic genes collagen type IV and fibronectin (Figures 5A to 5C). To investigate collagen and PAS-positive material deposition in the renal tissues we respectively performed Masson’s trichrome and PAS stainings on the renal sections. It was found that neither collagen depositions nor PAS-positive material accumulations were decreased after treatment with 10 mg/kg EMP in the renal sections of diabetic rats (Figures 6A to 6C).

***Renal cytoplasmic HMGB1, RAGE, and TLR-4 levels***

Renal levels of cytoplasmic HMGB1, TLR-4, and RAGE were measured by Western blotting. A significant rise in HMGB1, TLR-4, and RAGE levels were noticed in the control diabetic rats that were significantly alleviated by 4-week EMP treatment (Figures 7A to 7C). Moreover, EMP markedly reduced diabetes-associated upraise in the renal activities of NF-κB (Figure 7D).

## Discussion

In the present study, we demonstrated decreased levels of HMGB1, RAGE, and TLR-4 in the renal tissues of diabetic rats after 10 mg/kg EMP treatment for 4 weeks. These reductions were accompanied by the decline in the NF-κB activities, attenuations in the gene expressions of pro-fibrotic and pro-inflammatory cytokines, i.e., TGF-β, fibronectin, TNF-α, MCP-1, CXCL12, and RANTES, amelioration of oxidative stress markers including MDA, GPX and SOD, alleviation in the apoptosis marker caspase-3, reductions in the urinary indices of renal inflammation markers IL-6 and AGP, and finally improvements in the renal function as indicated by the kidney function tests, i.e., serum urea and creatinine levels.

EMP is a relatively new FDA-approved agent for the management of type 2 diabetes (13), and recent findings have proved its efficacy in the management of type 1 diabetes as an adjunctive therapeutic agent added to insulin (14). Additionally, EMP has been found to be a pleiotropic agent possessing anti-inflammatory and anti-oxidative stress properties that make it a potential renoprotective drug (15).

In addition to ameliorating renal inflammation as shown by mitigated NF-κB activities and IL-6 levels, EMP reduced glomerular hyperfiltration and urinary albumin excretions in the Akita mice model of type 1 diabetes (16). Furthermore, EMP increased renal heme oxygenase-1 (HO-1) levels in the same study, an eminent anti-oxidant protein downstream to the Nrf2 signaling pathway (16). It has been suggested that EMP may exert its anti-inflammatory and anti-oxidative stress effects through the inhibition of the receptor for advanced glycation end-products (RAGE) (10); Moreover, they showed that 4-week 10 mg/kg (p.o.) EMP decreased urinary levels of 8-hydroxydeoxyguanosine (OHdG), the marker of renal oxidative stress in STZ-induced diabetic rats; urinary excretions of albumin, however, remained unchanged in their investigation (10). In accordance with these findings, we demonstrated decreased gene expressions of fibrotic and inflammatory mediators (TGF-β, fibronectin, TNF-α, MCP-1, CXCL12, and RANTES). While protein levels of the inflammatory cytokines were not measured in the renal tissues, we observed that 4-week EMP treatment reduced urinary markers of kidney inflammation, i.e., IL-6 and AGP. Apart from being an inflammation indicator, AGP is the marker of renal endothelial dysfunction and yet is an especially sensitive marker for renal injury as it predicts future development of microalbuminuria in diabetic patients (17, 18). In addition, 10 mg/kg EMP alleviated oxidative stress in the renal tissues of STZ-induced diabetic rats as demonstrated by diminished levels of MDA and elevated activities of SOD and GPX in our study. Furthermore, we observed decreased gene expressions of pro-fibrotic cytokine TGF-β and fibrotic genes fibronectin and collagen type IV in diabetic kidneys after treatment with EMP; this result is consistent with the findings of previous investigations that showed anti-fibrotic effects for EMP against diabetes-associated renal fibrosis in db/db mice (11). Conversely, we observed no significant changes in the PAS-positive material and collagen deposition in the kidneys. These findings, however, seem to be conceivable because of the short duration of the study (3 months) not allowing gross renal fibrosis develop; it should, additionally, be underlined that the indices of renal fibrosis, i.e., TGF-β, collagen type IV, and fibronectin were detected to be decreased at gene expression levels.

HMGB1, a DNA-binding protein in the cell nucleus, is engaged in diverse biological functions such as RNA transcription, DNA repair, and cell differentiation (19). Nevertheless, when discharged from the activated cells, HMGB1 acts as a vigorous inflammatory mediator inducing its biological actions through interaction with its surface receptors RAGE and TLR-4 (8). HMGB1 is mainly expressed in the nuclear fraction of the healthy cells; under diabetic conditions, however, it is highly expressed in the cytoplasm of the renal cells together with RAGE and TLR-4 receptors (20, 21). Additionally, *in vitro* studies on human endothelial cells have revealed that cells exposed to the high amounts of glucose substantially increased the expressions of TLR-4, but not TLR-2 and that its expressions were even intensified more when the cells were exposed to the recombinant HMGB1 in their culture medium (21). Based upon these observations, and considering the ability of EMP in suppressing renal RAGE levels in diabetic kidneys (10), we examined renal cytoplasmic HMGB1, RAGE, and TLR-4 levels as well as nuclear NF-κB activities and concluded that 4-week 10 mg/kg EMP treatment down-regulated renal levels of all three proteins and simultaneously suppressed NF-κB activities in the STZ diabetic rats. It appears likely that EMP alleviates glucose accumulation in the renal tubular cells and therefore attenuates the expressions of HMGB1 and RAGE/TLR-4, the events that ameliorate inflammation and oxidative stress via reduced activities of the NF-κB signaling pathway (8).

## Conclusion

The findings of this investigation highlight the anti-inflammatory and anti-oxidative stress properties of EMP in the kidneys of STZ-induced diabetic rats by demonstrating EMP benefits in down-regulating renal levels of principal pro-inflammatory mediator HMGB1 and its correspondent receptor TLR-4; the events that collectively result in improved renal function as indicated by the decreased levels of serum urea and creatinine. It should be underlined that EMP-related alleviation in intra-cellular glucose accumulation down-regulates the activities of the master pro-inflammatory pathway NF-kB in renal proximal tubular cells (22). Apart from reductions in the expressions of the conventional inflammatory molecules like tumor necrosis factor alpha (TNF-α) and interleukin-6 (IL-6), reduced activities of NF-kB lead to decreased expressions of TLR-4 and therefore the inflammatory processes are further suppressed by a positive feedback mechanism (23). Moreover, TLR-4 is involved in the regulation of Nrf2 signaling pathway (24), the principal transcription factor that augments antioxidant defense mechanisms by up-regulating enzymes like superoxide dismutase and glutathione peroxidase in the involved tissues (25).

The present investigation, however, had a number of limitations that need to be addressed. Firstly, the relatively limited period of the study, i.e., 12 weeks hindered the development of overt sclerotic processes in the kidneys and therefore the effect of EMP on this cardinal feature of DN could not be evaluated. Secondly, the single dose (10 mg/kg) treatment with EMP limits the conclusiveness of the mechanistic studies in the present study.

## Conflicts of Interest

All authors declare no conflict of interests.
